# Oat—an alternative crop under waterlogging stress?

**DOI:** 10.3389/fpls.2024.1386039

**Published:** 2024-06-11

**Authors:** Britta Pitann, Karl H. Mühling

**Affiliations:** Institute of Plant Nutrition and Soil Science, Kiel University, Kiel, Germany

**Keywords:** nitrogen, oat, phosphorus, waterlogging, yield

## Abstract

**Introduction:**

Waterlogging is one vast environmental constraint that limits crop growth and yield worldwide. Most major crop species are very sensitive to waterlogging, leading to enormous yield losses every year. Much is already known about wheat, barley or maize; however, hardly any data exist on oat and its tolerance against waterlogging. Thus, this study aimed to investigate if oats can be an adequate alternative in crop rotation under conditions of temporal submergence and if cultivar differences exist. Furthermore, this study was to test (1) whether yield was differently affected when stress is applied at different developmental stages (BBCH 31 and 51), and (2) nutrient imbalances are the reason for growth restrictions.

**Methods:**

In a large-scale container experiment, three different oat varieties were cultivated and exposed to 14 consecutive days of waterlogging stress at two developmental stages.

**Results:**

Even though vegetative growth was impaired after early waterlogging and which persists till maturity, mainly due to transient nutrient deficiencies, growth performance after late waterlogging and grain yield of all three oat varieties at maturity was not affected. A high tolerance was also confirmed after late waterlogging in the beginning generative stage: grain yield was even increased.

**Discussion:**

Overall, all oat varieties performed well under both stress treatments, even though transient nutrient imbalances occurred, but which were ineffective on grain yield. Based on these results, we conclude that oats, independently of the cultivar, should be considered a good alternative in crop production, especially when waterlogging is to be expected during the cultivation phase.

## 1 Introduction

Climate change has been and is a serious topical global issue. While in the past the focus was mostly laid on the increase of climate-relevant gases, today, it is also important to understand the impact of hydrological changes. For example, in the course of climate change, an increase in the frequency and intensity of extreme weather events is to be expected, which threatens not only the security of the water supply but also food production as such. According to the Intergovernmental Panel on Climate Change (IPCC, 2022), almost half of the world’s population is already particularly affected by water scarcity. However, also the opposite is to be expected with more phases of heavy rainfall events, resulting in an increased risk of flooding associated with temporary waterlogging. Climate models already show that the global amount of precipitation increases by approximately 2% for every one-degree increase in temperature (Kreienkamp et al., 2016).

According to actual estimates, approximately 12% of the world’s arable land is currently at risk of waterlogging, and this is being exacerbated by unfavorable soil conditions (e.g., high clay content) and/or poor management systems (e.g., soil compaction and poor drainage) (Najeeb et al., 2015; Ploschuk et al., 2018; Alifu et al., 2022). Also, in Europe, the problem of waterlogging has long since arrived, with prolonged phases of heavy rainfall in winter and early spring, being more the rule than the exception (Deumlich and Gericke, 2020).

For Germany, it is undisputed that so-called heavy rainfall events have occurred more frequently over the past 15 years, at least regionally (Winterrath et al., 2017). This, in turn, has a vast effect on the agricultural sector, causing high yield losses of the major crops (e.g., Ploschuk et al., 2018).

Waterlogging induces several physiological changes in crops and thus affects various aspects of plant metabolism and growth (Horchani et al., 2009). These changes are primarily a response to a reduced availability of oxygen in the soil. Waterlogging as such is defined as the saturation of soil with water beyond its holding capacity (Striker, 2012). As a result, gaseous exchange with the atmosphere is inhibited, and gas diffusion in the soil is impeded (Jackson and Ricard, 2003), further driven as remaining oxygen is consumed by microbial activity. This lack of oxygen together with an increase in CO_2_ leads to anoxic soils (Ponnamperuma, 1972) and, consequently, results in severe hypoxia/anoxia within plant roots (Armstrong, 1980). This leads to root damage and decay, and also oxygen-depleted roots immediately shift from aerobic respiration to low ATP-yielding fermentation (Gibbs and Greenway, 2003). As a consequence, plants subsequently respond with stomata closure, which in turn reduces transpiration, a driver for water uptake and translocation. As an inevitable result, also nutrient uptake and translocation are restricted (Jackson and Drew, 1984; Colmer and Voesenek, 2009; McDonald, 2021), which may be further exacerbated by a shift of redox potential toward more reducing conditions. Together with the hampered gas exchange at the stomata and thus CO_2_ uptake, also photosynthesis is reduced, which in combination with restricted nutrient uptake leads to a marked decrease in plant biomass production and yield (Ashraf, 2012; Shao et al., 2013; Voesenek and Sasidharan, 2013; Arguello et al., 2016).

Depending on plant species, physiological tolerance, timing, and duration of the waterlogging event, yield losses can largely vary (Setter and Waters, 2003; de San Celedonio et al., 2014; Arduini et al., 2016; Ploschuk et al., 2018). Notably, high-yielding crops such as wheat or rapeseed are more susceptible to waterlogging in later developmental stages (Araki et al., 2012; Wollmer et al., 2018a, b; Hussain et al., 2022, 2023). According to Pampana et al. (2016), the yield of durum wheat was not affected when waterlogging occurs at the three- to four-leaf stage, which is also in line with de San Celedonio et al. (2014), who reported that wheat and barley are more sensitive at anthesis compared to tillering. This contradicts the results of Wu et al. (2015) and Ghobadi and Ghobadi (2010), who found that wheat was more prone to waterlogging when stressed at the seedling stage compared to later growth stages. However, there is a great consensus that the longer the waterlogging event persists, the greater the yield loss (Ghobadi and Ghobadi, 2010; Zhang et al., 2016; Tian et al., 2020).

Oats (*Avena sativa* L.) are among the food crops that rank sixth regarding cereal production right after wheat, maize, rice, barley, and sorghum (Ruja et al., 2021). Although being displaced by higher-yielding energy and protein crops in the past (Hoffman, 1995), today, oats are experiencing a revival as “super food” owing to their nutritional composition, and their production is gaining popularity again. Oats are well known for their versatility, thus tolerating a wide range of climatic conditions (Welsh, 1995; Ruja et al., 2021). However, while yield performance under waterlogging of the major crops has been well documented, studies on the response of oats to waterlogging are still scarce. However, there are indications that oats show a higher agronomic tolerance; i.e., they have the capability to maintain yields despite facing waterlogging during their growth cycle (Arduini et al., 2019). Watson et al. (1976) and Cannell et al. (1985) suggested that the better recovery potential of oats may be due to their capability to stay green during waterlogging and higher tiller fertility at maturity (Setter and Waters, 2003).

Based on these early findings and a lack of information, this study aims to investigate whether oats can be used as an alternative crop, especially under the changing climatic conditions present in Northern Germany. To gain further knowledge about possible cultivar variations, three oat varieties, namely, black oats, white oats, and yellow oats, were compared to facilitate cultivar choice on waterlogging-affected sites. Thus, it is hypothesized that 1) oats growth performance is less affected by waterlogging at later compared to earlier growth stages, 2) different oat varieties show no differences in growth performance and yield formation upon waterlogging, and 3) waterlogging-induced nutrient deficiencies are not yield-effective in oats.

## 2 Materials and methods

### 2.1 Plant cultivation and SPAD measurements

The experiment was conducted in the outdoor area of the Experimental Station of the Institute of Plant Nutrition and Soil Science, Kiel University, Germany (54°20′50″N, 10°6′55″E) starting in March 2021. Three oat (*A. sativa* L.) varieties (obtained from Saaten Union, Niedersachsen, Germany), Zorro (black oat; *A. sativa* var. *nigra*), Symphony (white oat; *A. sativa* var. *alba*), and Apollon (yellow oat; *A. sativa* var. *aurea*), were grown to maturity in large-scale containers (height, 0.9 m; area, 0.16 m^2^; volume, 120 L; see also Hohmann et al., 2016) with a seeding density of 300 seeds per container, which were later thinned to 90 plants after emergence. As a substrate, a subsoil (Cambisol; IUSS Working Group WRB, 2015) from the experimental station “Hohenschulen” of Kiel University, Germany, and arable topsoil from the district of Ost-Holstein (Schleswig-Holstein, Germany) were selected (see details in **Table 1**).

**Table 1:** Physico-chemical properties of the soils.

	Subsoil^a^	Topsoil^b^
Soil type	sL	lS
pH (CaCl_2_)	5.5	6.1
Total N (g/kg soil)	<0.3	n.a.
Phosphorus (mg/100 g soil)	5.0	5.0
Potassium (mg/100 g soil)	4.0	7.9
Magnesium (mg/100 g soil)	8.1	5.1

a According to analysis by Institut Koldingen GmbH, Germany.

b According to analysis by AGROLAB Agrar und Umwelt GmbH, Germany.n.a., not applicable.

The containers were filled with air-dried and homogenized soil as follows (from bottom to top): 1) 20 kg gravel as a drainage layer, 2) 100 kg subsoil + sand (1:1, w/w), 3) 30 kg subsoil + topsoil (1:1, w/w), 4) 10 kg topsoil plus fertilizer according to standard application for oats [in kg/ha: 100 N (split into N1 prior to seeding and N2 at shooting stage), 55 P, 80 K]. Weed and pathogen control were applied as required.

Soil plant analysis development (SPAD) values were measured on the fifth leaf after waterlogging at BBCH 31 and on the flag leaf after stress treatment at BBCH 51 (Meier, 2001). An average of 10 readings per container was taken using a chlorophyll meter (SPAD-502, Konica Minolta Sensing Europe B.V., Wrocław, Poland).

### 2.2 Stress treatments

Soil moisture was maintained at 60% water-holding capacity (WHC) until treatments started. While the respective controls (W0) were watered at 60% WHC throughout the entire crop cycle, waterlogging (100% WHC) was imposed for a total of two consecutive weeks: 1) W1 = early waterlogging at BBCH 31 and 2) W2 = late waterlogging at BBCH 51. Water treatment was checked every 2 days, and re-irrigation was performed based on weight loss if necessary. After terminating waterlogging, water was drained to achieve a target WHC of 60%, which was then retained until harvest. The experiment was set up with four replicates per treatment and oat variety in a completely randomized design (CRD). Randomization of the position of containers was performed twice a week together with the check of WHC.

### 2.3 Plant sampling and analysis

Two weeks after terminating waterlogging (W1 and W2), 30 plants (including side shoots) were randomly selected and harvested, and fresh weights were recorded. At maturity, the 30 remaining plants (including side shoots) per container were harvested and separated into straw and panicles. Subsequently, the biomass of straw and panicles, grain yield, and yield parameters were quantified. Panicles per container were counted and hand-threshed to determine total grain and thousand kernel weight. The number of grains per panicle was calculated as follows (Equation 1):


(1) $$Grains\;per\;panicle=\frac{Total\;grain\;weight÷thousand\;kernel\;weight\times 1,000\;}{Panicles\;per\;container}$$

To record dry weights, samples of each treatment were oven-dried at 60°C to constant weight and subsequently milled (Cyclotec 1093, Foss Tecator, Höganäs, Sweden) to fine powder for further analysis.

For mineral nutrient analysis, 200 mg of finely ground plant material of each plant part per replicate was digested with 10 mL 69% HNO_3_ in a microwave oven (1800 W, MARS 6, Xpress, CEM, Matthews, MC, USA) at 190°C for 45 min and subsequently analyzed by inductively coupled plasma–mass spectrometry (ICP-MS; Agilent Technologies 7700 Series, Böblingen, Germany) according to the method described by Jezek et al. (2015).

Determination of total N was conducted using a CNS elemental analyzer (Flash EA 1112 NCS, Thermo Fisher Scientific, Waltham, MA, USA), for which 5–10 mg of finely ground plant material was weighed into tin capsules. Results were validated using a certified wheat flour standard (Isotopenstandard Weizenmehl, IVA Analysentechnik, Meerbusch, Germany) as a reference.

### 2.4 Statistical analysis

Data were statistically analyzed using SPSS software (version 25.0). The analysis was based on four replicate containers per treatment set up as CRD. The effects of treatments per cultivar were tested using one-way ANOVA according to Duncan’s (homogeneity of variance) or Games–Howell (heterogeneity of variance) multiple-range tests at *p* ≤ 0.05. Significant differences are indicated by different letters. The significance of the correlations was tested using two-tailed Pearson’s correlation coefficient at *p* ≤ 1%.

## 3 Results

### 3.1 Fresh weights and SPAD values

**Figure 1:**
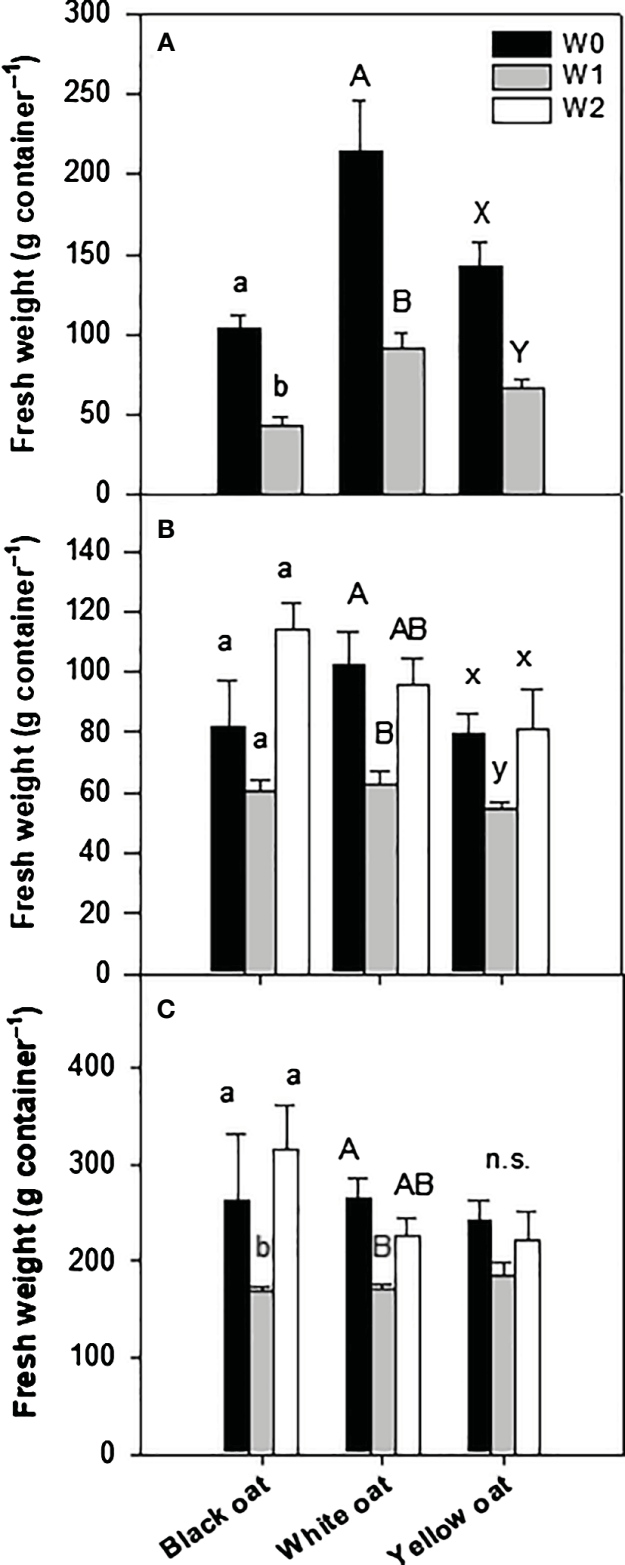
Fresh weight of the whole aboveground plant material after 14 days of waterlogging **(A)** at BBCH 31 and **(B)** at BBCH 51, as well as **(C)** at harvest after maturity. Bars represent means + standard errors (*n* = 4). Different letters refer to significant differences (*p* = 0.05; n.s., non-significant) between waterlogging treatments always within one oat variety. W0, control; W1, waterlogging at BBCH 31; W2, waterlogging at BBCH 51.

After 14 days of waterlogging at BBCH 31 (W1), all oat varieties clearly showed stunted growth and beginning chlorosis at older leaves (see **Supplementary Figure 1**). All oat varieties were similarly affected and showed a significant reduction in fresh weight of 58%, 57%, and 53% for black, white, and yellow oats, respectively (**Figure 1A**). Except in white oats, dry weight was not significantly reduced compared to the corresponding non-stressed control (data not shown).

At the second sampling date, 1 week after the late waterlogging event (W2), differences in the recovery potential between varieties became obvious (**Figure 1B**). While black oats were able to recover from early waterlogging, white and yellow oats still showed significantly impaired growth 6 weeks after water drainage. Interestingly, late waterlogging at BBCH 51 had no negative effect on the total fresh weight of all oat varieties and could maintain weights similar to the corresponding control (**Figure 1B**).

These results were largely confirmed at the final harvest (**Figure 1C**). While the recovery of white oats after the early waterlogging was not confirmed till maturity, fresh weight increased to the level of control for black and yellow oats (**Figure 1C**). However, the fresh weight of all oat varieties remained unaffected by late waterlogging.

**Figure 2:**
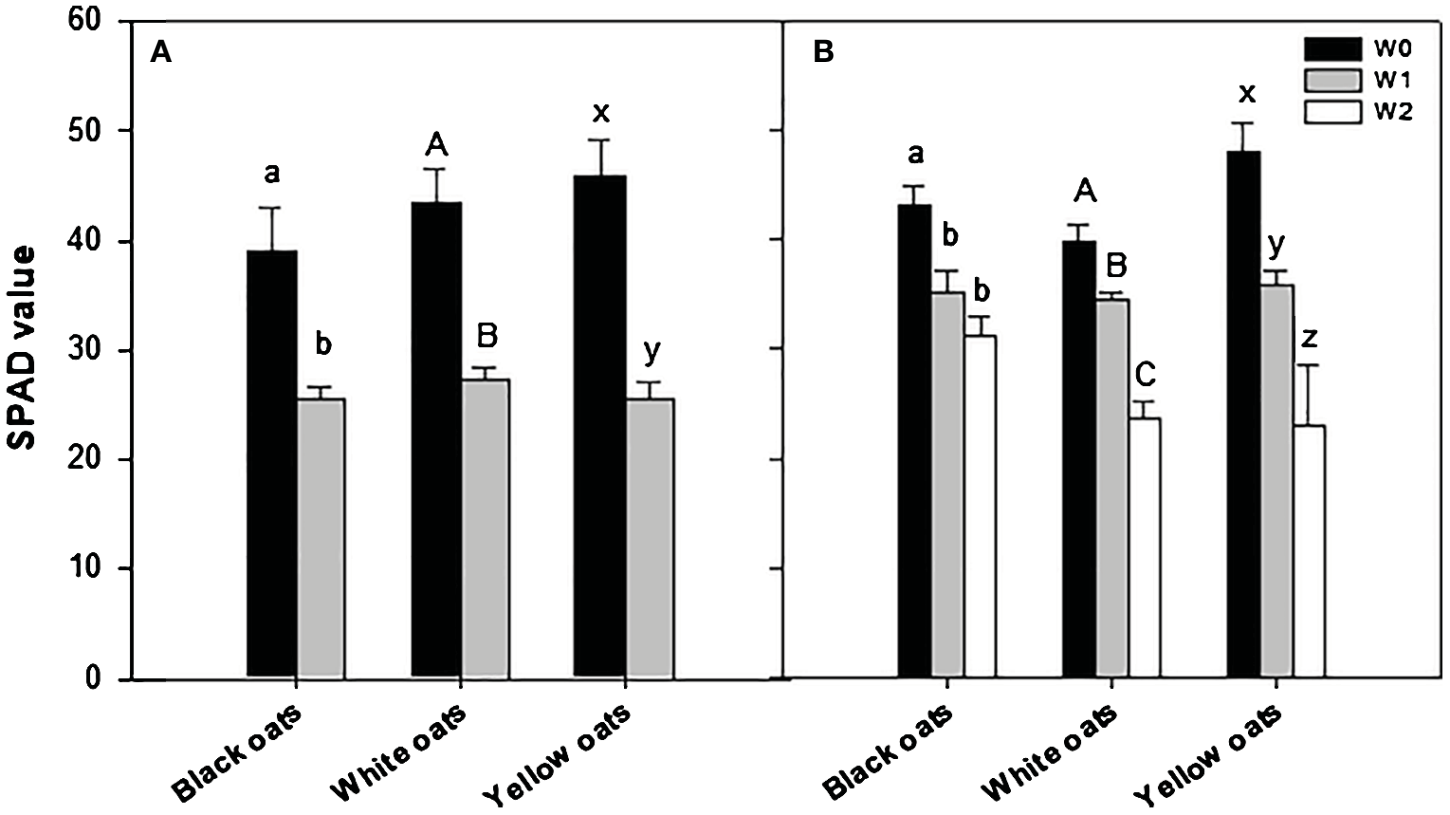
SPAD values after waterlogging **(A)** at BBCH 31 and **(B)** at BBCH 51. Bars represent means + standard errors (*n* = 4). Different letters refer to significant differences (*p* = 0.05) between waterlogging treatments always within one oat variety. W0, control; W1, waterlogging at BBCH 31; W2, waterlogging at BBCH 51; SPAD, soil plant analysis development.

SPAD values were measured always right after the termination of the waterlogging treatment. Significant differences were monitored between control plants and plants waterlogged at BBCH 31 for all three oat varieties (**Figure 2A**; **Supplementary Figure 2**). This waterlogging-induced decline in SPAD values was even more pronounced after stress treatment at BBCH 51 for white and yellow oats when compared to early waterlogging and control, while SPAD values in black oats remained unaffected by late waterlogging (**Figure 2B**; **Supplementary Figure 3**).

### 3.2 Yield and yield parameters

**Figure 3:**
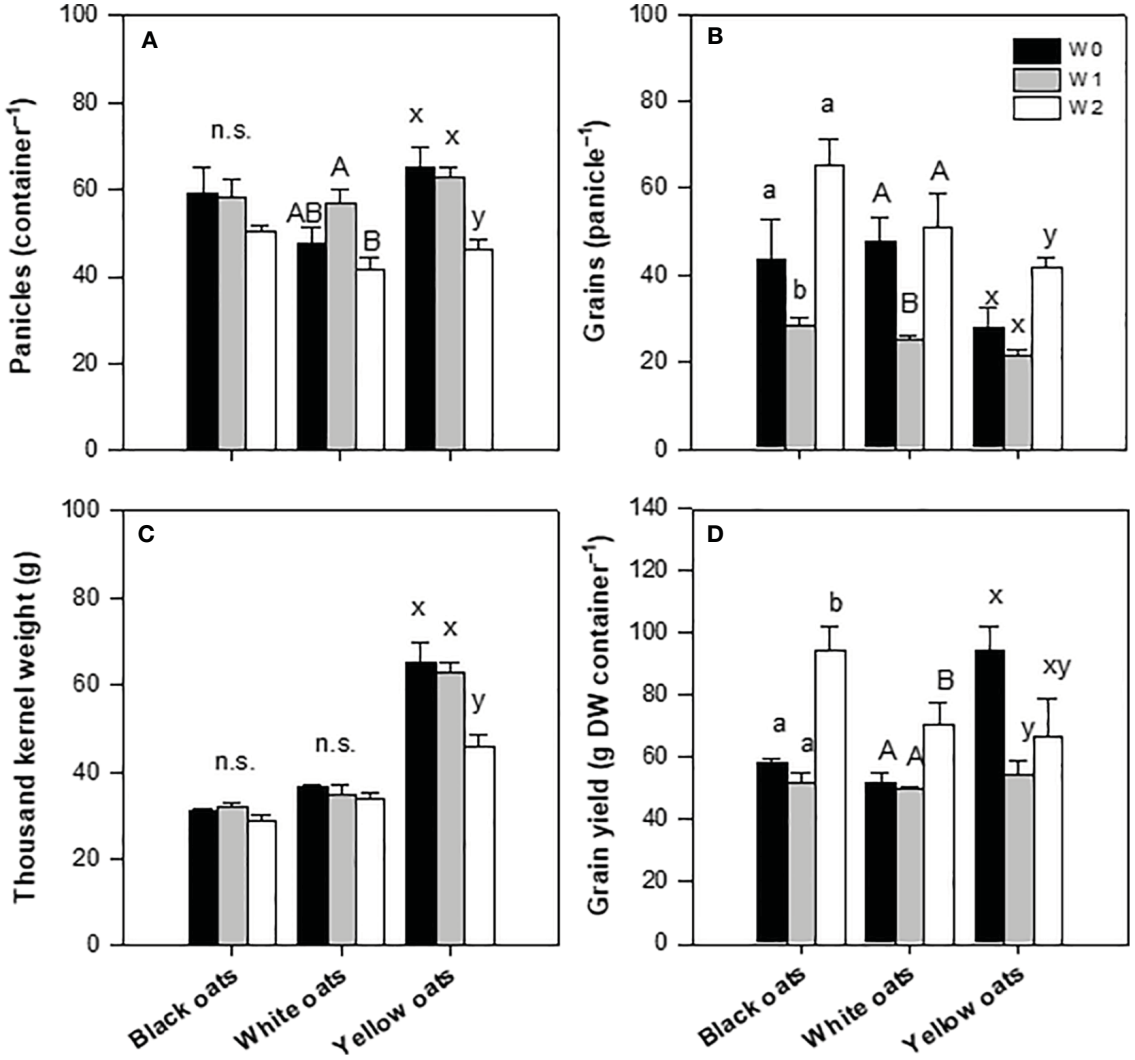
Yield components after harvest at maturity. **(A)** Number of panicles. **(B)** Grains per panicle. **(C)** Thousand kernel weight. **(D)** Grain yield. Bars represent means + standard errors (*n* = 4). Different letters refer to significant differences (*p* = 0.05; n.s. = non-significant) between waterlogging treatments within one oat variety. W0, control; W1, waterlogging at BBCH 31; W2, waterlogging at BBCH 51.

Whether the fresh weight was now broken down into the individual main major yield components, differences between oat varieties became obvious. While the number of panicles of black oats showed a slight but non-significant reduction at W1 and W2, white and yellow oats showed a significant reduction (**Figure 3A**). Concerning the number of grains per panicle, in black and white oats, W1 had a negative effect, leading to a reduced number of grains, while yellow oats were not influenced (**Figure 3B**). However, after W2, white oats compensated for the reduced number of panicles with the number of grains per panicle on the level of the control. Similarly, also, black and yellow oats significantly increased the number of grains after late waterlogging compared to W1 and remained on the level of their respective control (**Figure 3B**). Contrary to this, thousand kernel weight was not responsive at all to W1 and W2 in either black or white oats, while it was on a relatively high level in yellow oats compared to the other two varieties, but with a reduction after W2 (**Figure 3C**). This in turn led to an unchanged grain yield under W1 for both black and white oats, while under W2, there was even an increase in grain yield for both varieties (**Figure 3D**). However, yellow oats were the only variety that reacted sensitively to early waterlogging but could regain grain yield at least on the level of the control after late waterlogging (**Figure 3D**). Although differences in the major yield parameters were recognizable, the harvest index and the grain:straw ratio were unresponsive to both timings of waterlogging (data not shown).

Correlating yield with the single yield parameters showed that there was no correlation between yield and the number of panicles per container and thousand kernel weight for all three oat varieties, with all coefficients of determination being non-significant (**Supplementary Figures 3A–C, G–I**). However, yield significantly correlated to the number of grains per panicle at least for black oats (*R* ^2^ = 0.735; **Supplementary Figure 3D**) and white oats (*R* ^2^ = 0.779; **Supplementary Figure 3E**). Only yellow oats lacked such a correlation between yield and number of grains (*R* ^2^ = 0.118, **Supplementary Figure 3F**).

### 3.3 Nutrient concentration in plant tissues

**Figure 4:**
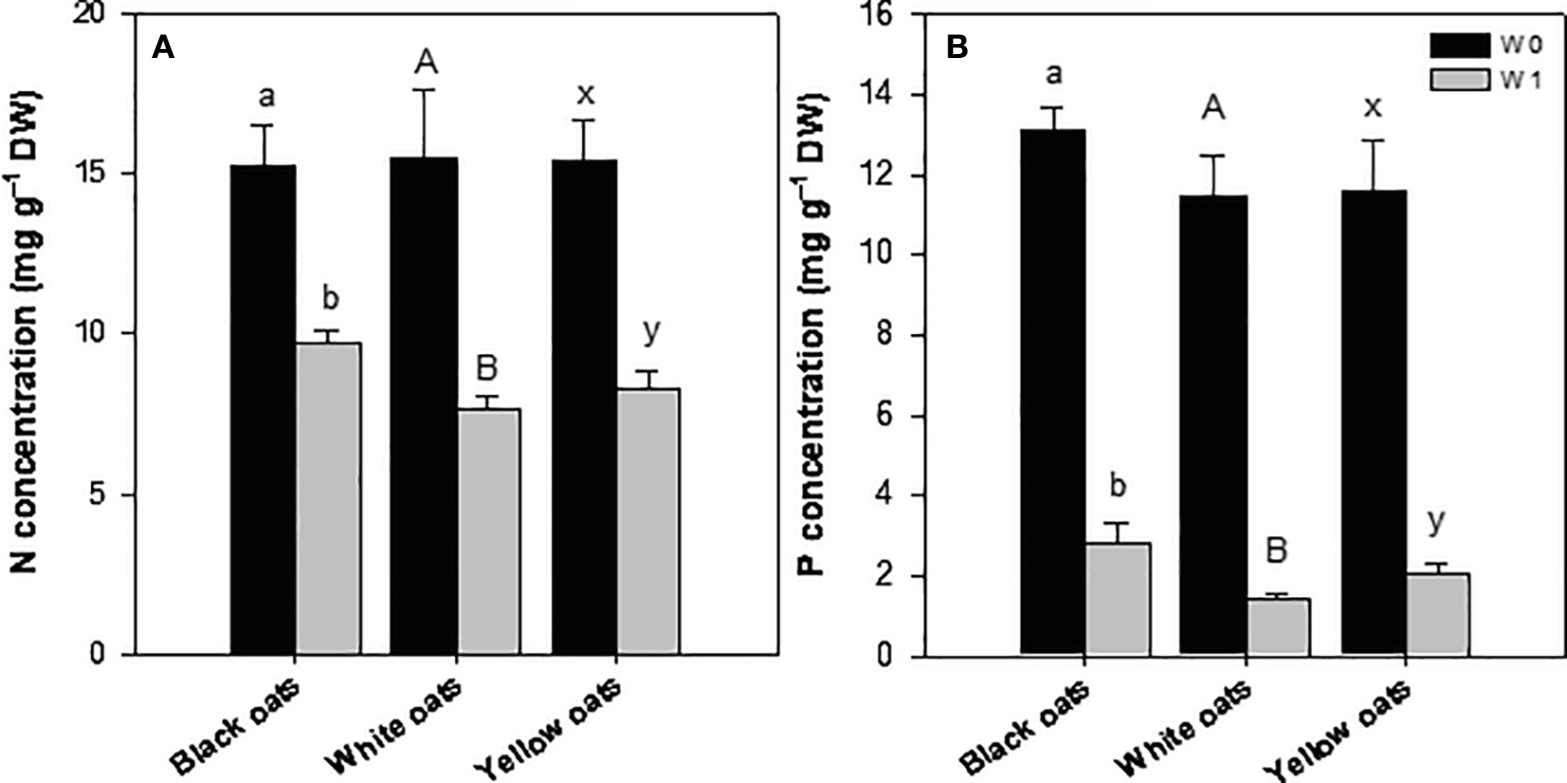
**(A)** Nitrogen and **(B)** phosphorus concentration of the whole aboveground plant material after 14 days of waterlogging at BBCH 31. Bars represent means + standard errors (*n* = 4). Different letters refer to significant differences (*p* = 0.05; n.s., non-significant) between waterlogging treatments within one oat variety. W0, control; W1, waterlogging at BBCH 31.

Early waterlogging (W1) resulted in a reduction of nitrogen (N) concentration in all three oat varieties. Hereby, the decrease was the highest in white oats (55%) followed by yellow oats (44%), and the lowest was in black oats (36%) (**Figure 4A**). Similarly to N, all oat varieties showed a strong decline in phosphorus (P) concentration, with white oats being most responsive compared to yellow oats and black oats (**Figure 4B**). Additionally, also S showed a marked decrease after early waterlogging (data not shown).

**Figure 5:**
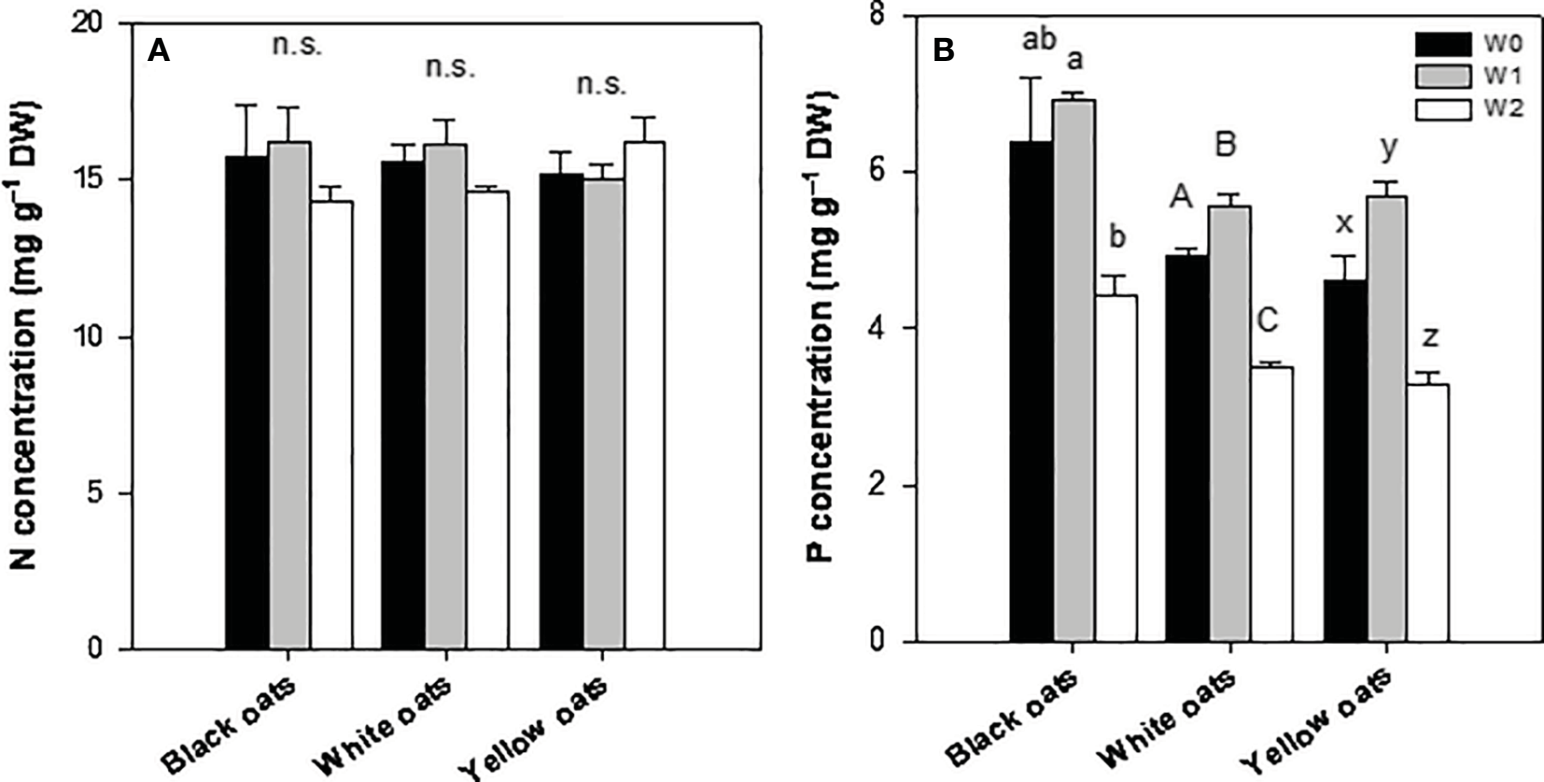
**(A)** Nitrogen and **(B)** phosphorus concentration of the whole aboveground plant material after 14 days of waterlogging at BBCH 51. Bars represent means + standard errors (*n* = 4). Different letters refer to significant differences (*p* = 0.05; n.s., non-significant) between waterlogging treatments within one oat variety. W0, control; W1, waterlogging at BBCH 31; W2, waterlogging at BBCH 51.

After late waterlogging, all oat varieties were able to recover, showing a N concentration similar to their respective control (**Figure 5A**). Furthermore, no effect of W2 on shoot N concentration could be determined. Similarly, the P status could be restored to the control level (black oats) or even increased (white oats and yellow oats) till BBCH 51 (**Figure 5B**). However, similar to early waterlogging, W2 led to a significant decrease in P in all three oat varieties (**Figure 5B**).

**Figure 6:**
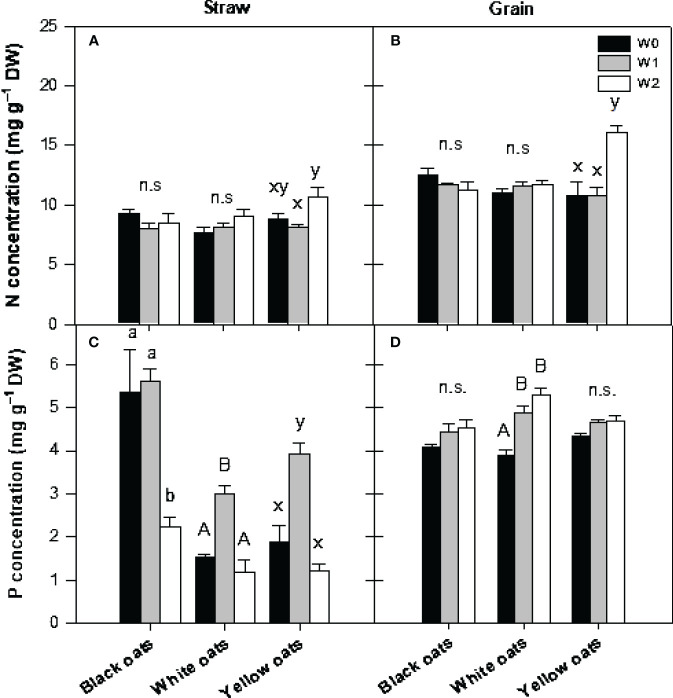
**(A, B)** Nitrogen and **(C, D)** phosphorus concentration in straw **(A, C)** and grains **(B, D)** after harvest at maturity. Bars represent means + standard errors (*n* = 4). Different letters refer to significant differences (*p* = 0.05; n.s., non-significant) between waterlogging treatments within one oat variety. W0, control; W1, waterlogging at BBCH 31; W2, waterlogging at BBCH 51.

At the timepoint of maturity, all plants were harvested, and nutrient concentrations in straw and grains were determined. With respect to straw N, it was observed that both black oats and white oats showed no changes in N concentration (**Figure 6A**). Only in yellow oats was a significant difference between W1 and W2 measurable, whereas no significant difference between W2 and the respective control was obvious. Likewise, also in grains of black and white oats, no effect of either W1 or W2 could be detected on N concentration (**Figure 6B**). However, yellow oats showed an increase in N at maturity when waterlogged at BBCH 51.

A similar pattern was measured for P concentration. Black oats were unable to regenerate the straw P level at W2, while in white and yellow oats, P concentration increased to the level of the well-drained control (**Figure 6C**). However, in grains of black and yellow oats, no waterlogging effect was measured (**Figure 6D**), but a significant increase in P in white oats at W1 and W2.

Furthermore, N and P concentrations in total shoot biomass as well as in grain at harvest were not effective on grain yield, with all coefficients of the determination being non-significant (**Supplementary Figure 4**).

## 4 Discussion

Globally, anthropogenic climate change intensified the risk of waterlogging, having multifaceted and severe impacts on economic and political pathways (IPCC, 2022), but also on crop production as such (Yang et al., 2017; Kaur et al., 2020b). Excess soil water has reduced rice, maize, soybean, and wheat yields by up to 50% annually (Hossain and Uddin, 2011; Ploschuk et al., 2018; Borgomeo et al., 2020; Ding et al., 2020; Tian et al., 2021). In Europe, according to actual estimates, flood-related risks and thus waterlogging will increase with a mean increment in annual output losses of approximately 11 million € per 1.5°C increase in global warming level (GWL) (Koks et al., 2019). However, are there any options to counteract such losses?

Identifying and breeding crop species being tolerant to waterlogging, in addition to other agronomical tools, can help mitigate the negative impact on crop physiology and improve overall agricultural resilience, especially in the long term (Kaur et al., 2020b). To date, many studies have focused on the major high-yielding energy and protein crops, such as wheat, oilseed rape, or maize. However, less is known about whether there are alternative crops that can be included in crop rotations and thus increase crop diversity when there is a risk of temporal waterlogging, which otherwise will delay farm operations (e.g., planting, fertilization, and harvest). Oats may represent one such alternative; that is why this study was conducted to evaluate the response of three different oat varieties to temporal waterlogging at two important developmental stages: shooting and panicle emergence. In order to simulate field-like conditions and to overcome limitations such as root growth restriction, which ultimately will affect nutrient uptake, large containers were chosen.

### 4.1 Growth performance and nutrient status of oats under temporal waterlogging

Waterlogged plants usually show wilting and development of chlorosis especially of the older basal leaves (Arbona et al., 2008). Also, Wollmer et al. (2018a) reported chlorosis formation on older leaves and even spot necrosis in winter wheat, which they explain as the oxidation of cell membranes by reactive oxygen species (ROS) formation and their reduced detoxification under waterlogging (Tan et al., 2008). Generally, chlorophyll reduction can be accredited to oxygen deficiency-induced changes in plant metabolism, promoting overproduction of ROS, mainly H_2_O_2_, and thus photooxidative damage of chloroplast (Yordanova et al., 2004; Zhang et al., 2015; Ren et al., 2016; Hasanuzzaman et al., 2017). As a consequence, photosynthesis will be decreased and thus biomass production (Zeng et al., 2020; Pais et al., 2023). However, even though SPAD measurements confirmed a decrease in chlorophyll in this study after early waterlogging (**Figure 2**), all tested oat varieties showed no distinct chlorosis but slightly brighter color compared to non-stressed plants (see **Supplementary Figure 1**). This is in contrast to wheat or barley, as oats have the capability to become less chlorotic and stay green even under waterlogged conditions, which gives an advantage to tolerate transient water stress (Setter and Waters, 2003). However, even though reduced biomass production under waterlogging is associated with lower photosynthetic activity (Ashraf, 2012), it is more likely a consequence of disturbed water and mineral uptake, rather than a photosynthesis effect (Colmer and Greenway, 2010; de San Celedonio et al., 2017).

As other crops (e.g., de San Celedonio et al., 2014; Ploschuk et al., 2018; Arduini et al., 2019; Hussain et al., 2022), oats also respond with an initial reduction in shoot growth especially when waterlogged in an early developmental phase (**Figure 1A**; **Supplementary Figure 1**). In agreement with Watson et al. (1976), growth reduction in oats under waterlogging was more pronounced when applied in an early growth stage and must be ascribed to a reduced or damaged root system. Notably, a loss in seminal roots and death of seminal root apical meristem were described, e.g., wheat (see Herzog et al., 2016). Ploschuk et al. (2023) also showed that root mass density was significantly reduced after early waterlogging in wheat, barley, oilseed rape, and pea, triggered by a lack of oxygen and the formation of ethylene. Likewise, also in oats, an initial decrease in shoot dry weight of 40% was explained by a decline in root dry weight of 50% (Watson et al., 1976; Cannell et al., 1985).

Hampered root growth is always accompanied by a decline in nutrient uptake, subsequently contributing to growth reduction. This effect is further triggered by a drop of redox potential and changes in pH in soil, also affecting nutrient transformation and availability, i.e., N and P (Patrick and Mahapatra, 1968; Hasanuzzaman et al., 2017, and literature within; Kaur et al., 2020b, and literature within). Such an effect is also shown in this study: nitrogen concentration dropped in W1 plants in all oat varieties (**Figure 4A**), corresponding to SPAD data (**Figure 2**), indicating a transient undersupply in the shooting stage. These results are in agreement with Arduini et al. (2019) for oats, Ren et al. (2017) for maize, Zhou et al. (1997) for oilseed rape, and Wollmer et al. (2018a) for wheat. In soil, nitrogen concentration, i.e., nitrate, will be decreased by several processes under waterlogging, such as runoff, denitrification, or nitrate leaching (see Kaur et al., 2020b, and literature within). However, decreasing nutrient concentrations in the vegetative shoot tissues can be explained not only by reduced root growth but also by inhibited uptake mechanisms. As plants switch to anaerobic respiration, they lack ATP, a necessity to drive ion uptake and xylem loading mediated by H^+^-ATPases (Colmer and Greenway, 2010; Elzenga and van Veen, 2010). Such decline in N concentration was not prominent in W2 plants (**Figure 5A**), which is attributed to the N fertilization (see Section 2.1) performed right after drainage of W1. This N dose served as a “post-waterlogging rescue N fertilizer” (Watson et al., 1976; Rasaei et al., 2012; Kaur et al., 2020a), leading to a regeneration of N status, which could be maintained until maturity in both straw and grain (**Figure 6A**). However, among all temperate cereals, oats must be considered as the crop with the greatest ability to regenerate from waterlogging (Watson et al., 1976; Cannell et al., 1985; Solaiman et al., 2007). Setter and Waters (2003) suggested that this is due to an extensive formation of aerenchyma, which coincides with increased root porosity (Herzog et al., 2016). Also, Solaiman et al. (2007) described an increase in root porosity from 6% (well drained) to approximately 20% (waterlogged) in adventitious roots of oats compared to 2% in seminal roots. By this, O_2_ in roots is kept high, allowing the roots to maintain aerobic respiration and high ATP levels and thus improve nutrient uptake characteristics (Colmer and Greenway, 2010; Takahashi et al., 2014).

As the redox potential drops, the solubility of P increases due to a loss of sorption sites. This in turn leads to a higher pore water concentration (Patrick and Mahapatra, 1968) and thus plant availability and uptake. However, like N concentration, P concentration also declined at W1 (**Figure 4B**), indicating a period of deficiency. As this was not expected, it must be reasoned that 1) P either leached down (Smith, 2020; Rupngam et al., 2023), 2) P retention in soil was increased due to sorption and/or precipitation with free Fe (Patrick and Mahapatra, 1968; Smith, 2020; Rupngam et al., 2023), or 3) uptake is inhibited, as the limited internal energy under waterlogging is directed to internal pH regulations and transport of solutes involved in anaerobic respiration (Greenway and Gibbs, 2003). This effect was reversed at W2 for all oat varieties (**Figure 5B**), indicating a full regeneration of the P status in W1 plants, which can be ascribed to an increased uptake due to higher available P and uptake in submerged soils. However, submergence at W2 again led to an undersupply of P in all three oat varieties. However, these were only of a transient nature in white and yellow oats (**Figure 6C**). Even though it seems that the time span till maturity was not enough for full recovery in black oats, a dilution effect must be assumed, as P content was on the level of control for all waterlogging treatments in all oat varieties (data not shown), though grains were not affected at all by all waterlogging events (**Figure 6D**). This is in line with Cannell et al. (1985), who also found no differences in N and P concentrations at harvest between treatments.

### 4.2 Yield response of oats under early and late waterlogging

Although much research was conducted on various crops, such as wheat (e.g., de San Celedonio et al., 2017; Ploschuk et al., 2018; Wollmer et al., 2018a; Pais et al., 2023), oilseed rape (e.g., Wollmer et al., 2019; Hussain et al., 2023; Zhu et al., 2023), barley (e.g., Masoni et al., 2016; de San Celedonio et al., 2017), or maize (e.g., Tian et al., 2019; Liang et al., 2020), there are hardly any data available on oats’ response to waterlogging, especially regarding cultivar differences, the existence of flooding-related quantitative trait loci (QTLs), or -omics data on waterlogging and associated O_2_ deprivation (Mustroph, 2018).

While yield decreases in a range of a few percent up to an almost total loss are reported, a meta-study by Tian et al. (2021) revealed that approximately 3% of all database samples showed contrasting behavior, thus increasing yield. One explanation for this phenomenon is the capability of such crop varieties to tolerate time periods of waterlogging. Thereby, it plays a crucial role in which developmental stage waterlogging occurs and for how long crops are submerged. In this study, it was found that oats, for the most part, are characterized by a high tolerance to both early and late waterlogging. While the grain yield of black and white oats was unaffected by early waterlogging and even increased after late waterlogging (**Figure 3D**), only yellow oats were sensitive to early waterlogging. However, the grain yield reduction of yellow oats after late waterlogging was only slightly but non-significantly reduced (**Figure 3D**). Such high recovery rates, at least as shown for black and white oats, are also consistent with the few published data for oats. For example, Watson et al. (1976) showed that when waterlogging ceased, oats recovered better than, e.g., wheat or barley. They reported that especially ear emergence was more delayed in these crops, which was even more pronounced at very early waterlogging (2 weeks after seeding) or when seeding was already delayed, shortening the recovery phase and leading to less grain per ear. In contrast, similarly to waterlogging at BBCH 51 in this study, waterlogging 6 weeks after seeding or at ear emergence was of minor effect, which was also confirmed for winter wheat (Watson et al., 1976; Cannell et al., 1985). Also, other studies, e.g., on wheat, report that early reproductive states are more adversely affected than tillering stages (Setter and Waters, 2003). However, this contradicts the results of Wollmer et al. (2018a), who showed the highest yield reduction of wheat after waterlogging in the generative phase.

In oats, the by far largest reduction in grain yield was observed when plants were waterlogged at the tillering stage, caused by the formation of smaller grains. This effect was almost completely eliminated, when N was applied (Watson et al., 1976). This is in agreement with this study, in which the grain yield of W1 and W2 plants was similar or even increased in the case of black and white oats (**Figure 3**). Only in yellow oats was the speed of grain yield recovery somewhat slower but could reach a value comparable to the control after W2. Similar results were found for winter oats (Cannell et al., 1985), where tillering was reduced but could be reversed by N application. Reductions in tillers though were not found in this study at W1, rather than an increase for white oats (data not shown), probably compensation grain yield reductions. Only white and yellow oats at W2 showed a reduced number of panicle-bearing tillers, but this was also reversed and did not affect grain yield due to compensation by a distinct increase in grain number per panicle in yellow oats (**Figure 3**). A reduction in kernel weight of 9% in oats, as reported by Cannell et al. (1985), could thereby only be confirmed in yellow oats (**Figure 3**), while the other two varieties did not show any change compared to the well-drained treatment. In comparison, under similar conditions, for wheat, a reduction of 10%–30% was reported (Cannell et al., 1985; Ploschuk et al., 2018), indicating the high recovery potential of the tested oat varieties in this study. However, the observed reduction is not caused by a reduced number of grains per panicle rather than the reduced number of panicles in total.

In complete contradiction to the already discussed studies are data by Arduini et al. (2019). Similar to this study, oats were waterlogged at tillering after seeding in spring, differently from Watson et al. (1976), who used winter oats. This difference has of large effect on the regeneration period; while winter oats had a prolonged phase of 118 days after draining, the recovery phase in spring oats is much shorter. Therefore, Arduini et al. (2019) argued that higher temperatures of 20°C during waterlogging could in part be responsible for the higher sensitivity observed in their study. Although after 14 days of waterlogging a not yet significant decrease in harvest index became obvious, *A. sativa* compared to *Avena byzantina* showed a 79% and 83% reduction in grain yield, respectively, resulting in a decrease in harvest index of 8% and 10%, respectively, after 35 days of submergence (Arduini et al., 2019). Similarly, in the present study, the harvest index remained on the level of the respective controls for all three oat varieties (data not shown) at W1 and W2 after only 14 days of waterlogging, which may go back to an increased tiller fertility (Watson et al., 1976; Cannell et al., 1985).

## 5 Conclusions

Even though there are only limited data on oats’ response to waterlogging, it is obvious from the literature that a high diversity exists among different varieties. Thus, this study contributes to the understanding of the stress tolerance of oats and offers a solution to rethink established crop rotations, especially in the context of climate change and the associated risk of flooding/waterlogging in the future.

The oat varieties tested in this study, i.e., black, white, and yellow oats, are standard cultivars recommended in Germany due to their stable yield potential. All varieties differed slightly in their response to waterlogging, but all showed an initial decrease in fresh weights when waterlogged in the vegetative phase. This growth reduction was most probably caused by a transient deficiency in nitrogen and phosphorus; however, N deficiency was counteracted by a second N-fertilizer dose right after ceasing the stress, guaranteeing a proper N supply till maturity. Further, also, the P status recovered, although the oat varieties differed in the regeneration time, which may be attributed to the restoration capacity of the root system. Although all varieties were differently affected regarding yield components, i.e., number of panicles, grains per panicle, or thousand kernel weight, all oat varieties showed grain yields comparable to well-drained soil conditions or even higher in case of black and white oats, independent from the timing of the waterlogging stress. However, early waterlogging in the vegetative phase (BBCH 31) was more harmful in contrast to late waterlogging in the generative phase (BBCH 51), but all varieties were able to compensate till maturity. Thus, it is reasoned that oats, or at least the varieties used in this study, showed a high tolerance level to temporal submergence, which was not affected by waterlogging-induced nutrient deficiency.

Therefore, we conclude that oats represent a suitable alternative and can compete with high-yielding but more sensitive crops, such as wheat, especially on marginal sites with lower yield potential and sites that are prone to waterlogging in Northern Germany.

## Data availability statement

The original contributions presented in the study are included in the article/**Supplementary Material**. Further inquiries can be directed to the corresponding authors.

## Author contributions

BP: Writing – original draft, Writing – review & editing, Conceptualization, Investigation. KM: Conceptualization, Writing – review & editing.
