# Supplementary material for: Oat—an alternative crop under waterlogging stress?
Source: Front Plant Sci. 2024 Jun 11;15:1386039. doi: 10.3389/fpls.2024.1386039 (PMC11196775; doi:10.3389/fpls.2024.1386039)
Supplement: Supplementary file 1 [file DataSheet_1.pdf]

Supplementary Material

1 Supplementary Figures

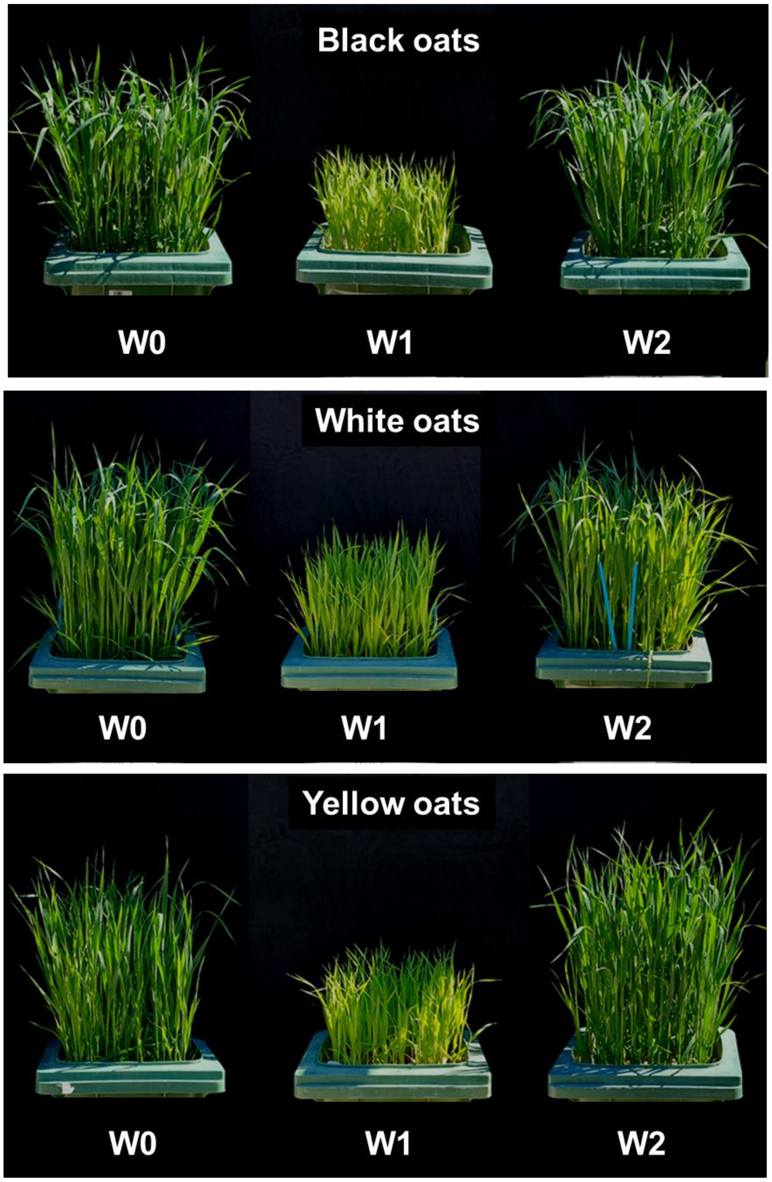

Supplementary Figure 1. Effect of early waterlogging (BBCH 31) on aboveground biomass on three different oat varieties. Abbreviations: W0 = control, W1 = waterlogging at BBCH 31, W2 = waterlogging at BBCH 51. Note, that W2 plants were not stress treated at this time point.

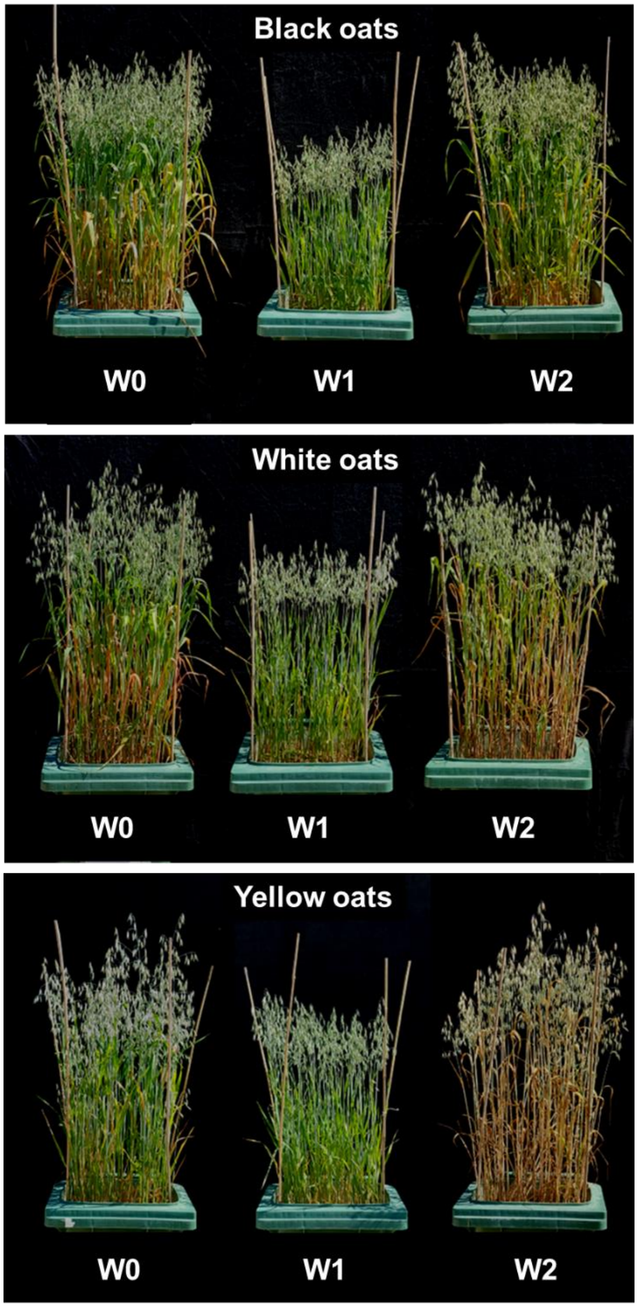

Supplementary Figure 2. Effect of late waterlogging (BBCH 51) on aboveground biomass on three different oat varieties. Abbreviations: W0 = control, W1 = waterlogging at BBCH 31, W2 = waterlogging at BBCH 51.

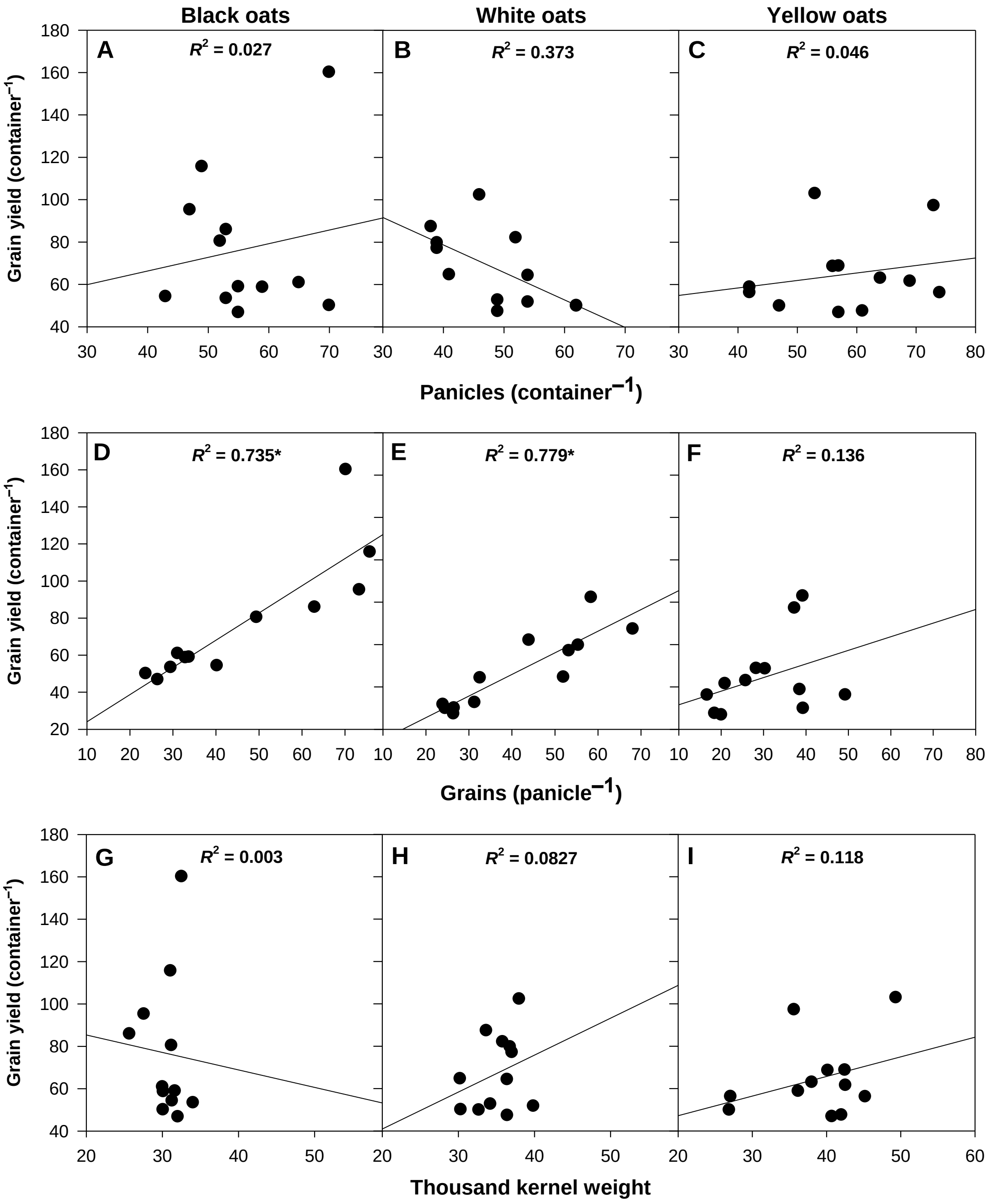

Supplementary Figure 3. Correlations between yield and (**A**, **B**, **C**) number of panicles, (**D**, **E**, **F**) grains per panicle, and (**G**, **H**, **I**) thousand kernel weight in black oats (left), white oats (center) and yellow oats (right). Asterisks (*$p \leq 0.05$) indicate significant coefficients of determination.

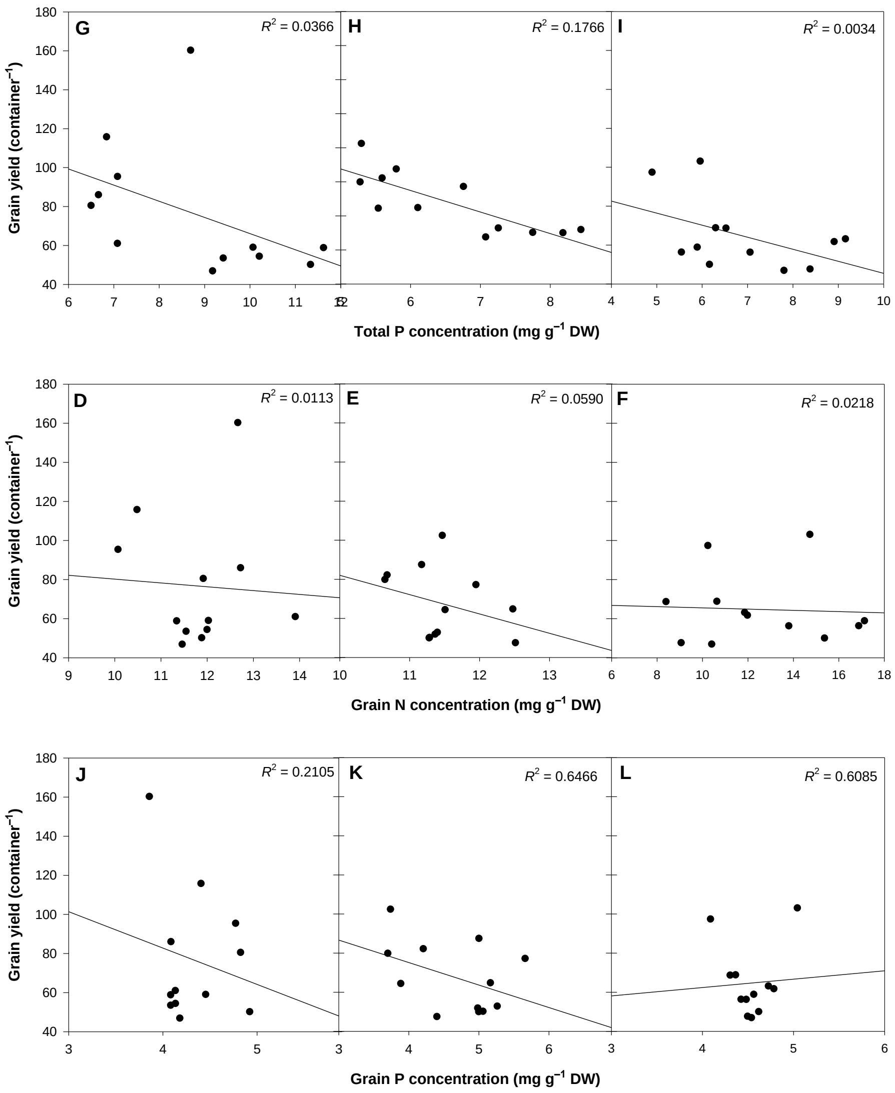

Supplementary Figure 4. Correlations between yield and (**A**, **B**, **C**) total N concentration, (**D**, **E**, **F**) grain N concentration, (**G**, **H**, **I**) total P concentration, and (**J**, **K**, **L**) grain P concentration in black oats (left), white oats (center) and yellow oats (right).
